# Early Experience in Da Vinci Robot-Assisted Partial Nephrectomy: An Australian Single Centre Series

**DOI:** 10.1155/2015/671267

**Published:** 2015-06-08

**Authors:** Francis Ting, Richard Savdie, Sam Chopra, Carlo Yuen, Phillip Brenner

**Affiliations:** St Vincent's Hospital, Darlinghurst, Sydney, NSW 2010, Australia

## Abstract

*Introduction and Objectives.* To demonstrate the safety and efficacy of the robot-assisted partial nephrectomy (RAPN) technique in an Australian setting. *Methods.* Between November 2010 and July 2014, a total of 76 patients underwent 77 RAPN procedures using the Da Vinci Surgical System© at our institution. 58 of these procedures were performed primarily by the senior author (PB) and are described in this case series. *Results.* Median operative time was 4 hours (range 1.5–6) and median warm ischaemic time (WIT) was 8 minutes (range 0–30) including 11 cases with zero ischaemic time. All surgical margins were clear with the exception of one patient who had egress of intravascular microscopic tumour outside the capsule to the point of the resection margin. Complications were identified in 9 patients (15.8%). Major complications included conversion to open surgery due to significant venous bleeding (*n* = 1), reperfusion injury (*n* = 1), gluteal compartment syndrome (*n* = 1), DVT/PE (*n* = 1), and readmission for haematuria (*n* = 1). *Conclusion.* This series demonstrates the safety and efficacy of the RAPN technique in an Australian setting when performed by experienced laparoscopic surgeons in a dedicated high volume robotic centre.

## 1. Introduction

Partial nephrectomy (PN) is now the most commonly performed surgery for renal cell carcinoma (RCC) [1], with the AUA and EAU guidelines recommending PN as the standard of care for masses 4–7 cm [2, 3]. PN for RCC has been shown to have lower morbidity and equal oncological outcomes to radical nephrectomy as long as pathological/surgical margins are clear of tumour [4].

Furthermore, robotically assisted partial nephrectomy (RAPN) has surpassed laparoscopic partial nephrectomy (LPN) in the United States as the more frequently performed minimally invasive surgery for RCC [1].

Most surgeons continue to perform PN via an open approach as even those who are very skilled at laparoscopy find LPN technically challenging with long clamp times [4], potentially causing ischemic renal damage in the longer term. Additionally, the technical challenges presented by LPN have possibly led to the overuse of laparoscopic radical nephrectomy when PN may have been feasible [4].

There is increasing evidence that overall health is compromised in the presence of reduced renal function associated with a complete nephrectomy, with increased risk of cardiovascular events and hospitalisation being major contributors to long-term morbidity [5].

Da Vinci RAPN allows precise excision, greater dexterity, and ease of suturing to assist in renorrhaphy. This results in much shorter periods of ischaemia and potentially less renal ischaemic damage [4, 6]. It facilitates greater technical proficiency which expands the scope of minimally invasive partial nephrectomy to include more complex lesions, including lesions larger than T1a, hilar lesions, and those with venous tumour thrombosis [6, 7].

## 2. Methods

Between November 2010 and July 2014, a total of 76 patients underwent 77 RAPN procedures using the Da Vinci Surgical System© at St Vincent's Private Hospital by a selected team of surgeons, anaesthetists, and scrub staff dedicated to refining this procedure. The senior author performed the majority of the cases and these 58 cases have been highlighted in this case series.

Initially, the series consisted of patients with small renal tumours (T1A) in a favourable position but, as the series progressed, patients with solitary kidneys (*n* = 5) and multiple tumours (*n* = 2) and those with small (<4 cm) but unfavourably located tumours were also included (*n* = 12).

Preoperative work up included a serum creatinine and haemoglobin, a 24-hour creatinine clearance, DTPA with differential function, and calculated estimated glomerular filtration rate (eGFR).

Postoperatively, serum creatinine was assessed at one month, two months, three months, and six months, with a further DTPA scan at six months. DTPA scanning was discontinued after the first 12 patients as the literature indicated that a serum creatinine at this time would give similar information and this was consistent with our findings.

At the beginning of the series, standard renal imaging was accepted but, as the series progressed, a dedicated CT scan with arterial phase images at 3 mm cuts became mandatory for assessment of renal vasculature.

Complications from the procedure were recorded and graded according to the Clavien classification system.

### 2.1. Operative Technique

Patients were given routine intravenous hydration, but no Lasix or Mannitol was given intraoperatively.

**Figure 1:**
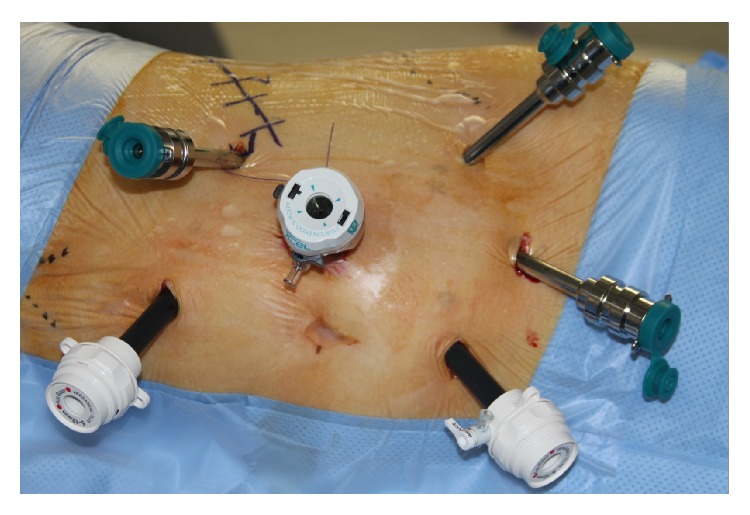
Port placement.

**Figure 2:**
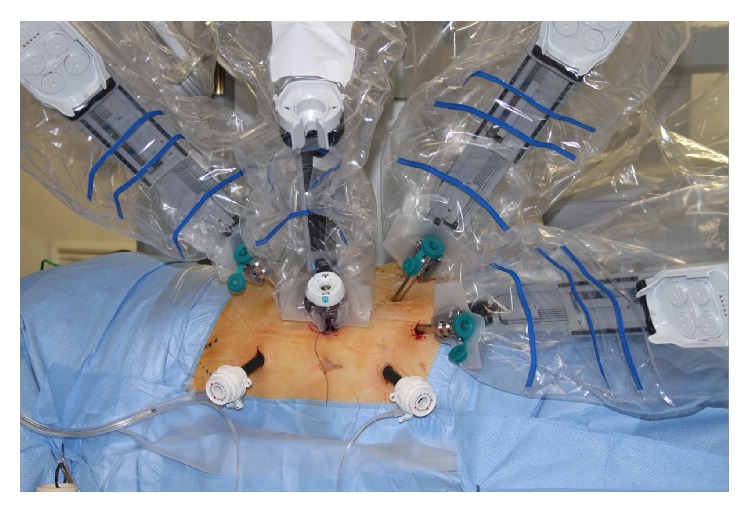
Port placement with Da Vinci robot.

Surgery was performed in standard flank position with usual attention to pressure areas. The anaesthetist placed an arterial line but not a central line as a routine. Ports were placed as shown in the diagram (Figures 1 and 2), with intermittent use of an extra port for the “Fourth Arm” when perceived to be of use. For right-sided tumours, it was occasionally necessary to place a 5 mm port for a liver retractor. Insufflation to achieve intraoperative laparoscopic pressures of between 6 and 8 cms H_2_O was utilised to minimise compromise to renal function.

All renal arteries and renal veins were isolated and controlled with a Vesi-loop© using the preoperative CT as an adjunct in defining the arterial anatomy. If possible, the segmental vessel supplying the tumour alone was selectively isolated. Prior to clamping the renal artery, special care was taken to confirm that multiple preprepared sutures for the repair were available and that the robotic needle-drivers were available and functioning. Clamping of the renal vessel was done with laparoscopic bulldog clips, applied by the assistant.

Early in the series both renal artery and renal vein were clamped; currently only the artery is clamped. Intra-abdominal pressure was raised to between 15 and 20 cms of water for a few minutes if venous bleeding was apparent.

Ultrasound localisation of the tumour was performed intraoperatively to mark out the resection margins on the surface of the kidney. The area around the tumour was marked at a distance of 1 cm with cautery once the kidney was mobilised. The renal artery was then clamped and the tumour was excised with cold scissors to allow good visualisation. If the tumour was visualised, extra tissue was taken from the deep margin. No frozen sections were performed.

The defect was then repaired with a running 2.0 vicryl across the base of the defect and then interrupted one-vicryl sutures spaced one centimetre apart. We utilised the technique of sliding clip renorrhaphy [8] where large Hem-O-Lok are applied and fastened with the LapraTy© clip. The ability to tighten the Weck Haem-O-Lock clips© and then fasten with the LapraTy© was essential for haemostasis. Flo-seal was used for minor ooze but no bolsters were used.

Early unclamping of the renal artery was performed where possible after placing the running stitch at the base and the first one to two interrupted one-vicryl repair sutures before completing the renorrhaphy.

The perinephric fat was closed around the defect once haemostasis was ensured and a drain was placed in all patients.

## 3. Results

Demographic and tumour data is summarised in Table 1 and perioperative data is summarised in Table 2.

The mean operative time was 4.2 hours (range 1.5 to 6 hours), with a 98.3% procedural success rate due to one case requiring conversion to open. Intraoperative estimated blood loss (EBL) was between 50 and 100 mLs for 46 patients (79.3%). Seven patients (12.1%) had EBL of 200 mL, four patients (6.9%) had EBL of 400 to 500 mLs, and one patient (1.7%) had EBL of 1000 mL. Only two patients received a blood transfusion. Massive transfusion was given to one of these patients for significant venous bleeding which required conversion to open nephrectomy. The other patient received two units of packed red blood cells which were given postoperatively in intensive care for Haemoglobin (Hb) of 86 g/L in the setting of known ischaemic heart disease (preoperative Hb of 113 g/L).

Overall, median warm ischaemic time (WIT) of eight minutes was achieved (range 0 to 30 minutes) by the senior author. It was noted that ischaemic times by the senior author dramatically improved as the series progressed. In the first ten patients, median WIT was 18 minutes. This improved to a median WIT of 4.5 minutes in the final 37 patients which included eleven patients with zero ischaemic time, that is, either no clamping or clamping of a third-order artery only (Table 3).

All surgical margins were clear with the exception of one who had egress of intravascular microscopic tumour outside the capsule to the point of the resection margin. The specimen was otherwise microscopically and macroscopically clear of the tumour bulk. All tumours were 80 mm or less, with a mean tumour size of 30.5 mm.

The pathology as expected from a group of mainly T1 patients was primarily clear cell renal cell carcinoma in 36 of the 58 (62.1%), papillary renal cell carcinoma in 8 (13.8%), renal oncocytoma in 7 (12%), chromophobe renal cell carcinoma in 4 (7%), two fat-poor angiomyolipoma (3.4%), and one malakoplakia (1.7%).

For the patients who did not fall into the solitary kidney group, at a minimum follow-up of three months all but three had creatinine within 20 micromol/L of preoperative levels. The first patient was early in the series and had a clamp time of thirty minutes. His postoperative DTPA showed loss of function in the left kidney. The second patient had a clamp time of only ten minutes but is presumed to have had reperfusion injury. Clinically, he developed pain and fever on day 1; angiogram showed normal vascularity and diagnostic laparoscopy was normal. The third had open conversion and nephrectomy.

In the five patients with solitary kidney, four of them maintained creatinine within 30 micromol/L of their preoperative level, including one who started with creatinine of 170 micromol/L.

Complications according to the Clavien classification system [9] are outlined in Table 4. Twelve postoperative complications were identified in nine patients (15.8%) with the remaining 48 patients (84.2%) being free of complications.

There were three Clavien Grade-3 complications. This included the previously mentioned reperfusion injury and also the patient who was discovered to have significant venous bleeding after completion of the partial nephrectomy, which required massive transfusion and conversion to open nephrectomy. The third patient was readmitted with haematuria two weeks postoperatively in the context of recommencing therapeutic Clexane for AF a few days before. Angiogram was unremarkable and the bleeding stopped spontaneously.

The commonest postoperative problem was atelectasis in three of the 57 patients (5.3%). One of these patients had pneumothorax (resolved without drain), and one patient required antibiotics for a pneumonia.

Gluteal compartment syndrome was diagnosed in one patient despite meticulous positioning. The patient was 135 kgs and recovered completely without operative intervention.

One patient suffered a deep venous thrombosis with pulmonary embolus, but in retrospect this had likely been present prior to the surgery but was not detected or treated.

Most patients had a length of stay between 4 and 8 days, with a median stay of six days and a range between 3 and 23 days (patient with DVT and pulmonary embolus). This reflected an older age group of patients with comorbidities rather than surgical complications.

## 4. Discussion

This paper demonstrates the safety and efficacy of the RAPN technique in an Australian setting. RAPN has overtaken LPN in the US as the most commonly performed minimally invasive procedure for PN [1]. Much like the uptake of robot-assisted prostatectomy in our country, RAPN is sure to follow.

The earliest reported feasibility study on RAPN was in 2004 by Gettman et al. [10] in the Mayo Clinic. Since this initial study, there has been a multitude of reports on RAPN, with significant reported advancements in technique and technology, and RAPN is fast becoming the technique of choice for most T1a renal tumours, with the caveat being that the appropriate equipment and expertise are available [11].

Two recent meta-analyses comparing RAPN to LPN have been recently published which have shown that operating times, estimated blood loss, conversion rates, positive surgical margin rates, and complication rates are similar for both groups but with the advantage of significantly shorter WIT in RAPN compared to in LPN [4, 12]. The learning curve in LPN has been shown to be much steeper, with mastery of the procedure performed under warm ischaemia requiring completion of more than 500 cases [13], compared with much smaller numbers in RAPN of about 30 cases or fewer [11]. This has been complemented by the present series in which the median WIT of 12.5 minutes for the first 21 cases drastically dropped to almost a third of this to a median of 4.5 minutes for the last 37 cases for the senior author. However, it should be noted that many of these tumours were carefully selected and that the primary surgeons are experienced laparoscopic and robotic surgeons with a combined laparoscopic renal procedure history of over one thousand cases in a high volume tertiary referral centre, which would have had a considerable impact on the initial learning curve.

Selective segmental clamping was performed on nine of the patients in this series and no clamping of renal artery was performed in two patients with procedural success in all cases thus achieving avoidance of global renal ischaemia in 11 patients.

In our series, intraoperative ultrasound was critical in noting the deep extent of the tumour, particularly as the tumours always seemed to be deeper than estimated due to the magnified view at the surgeon console. Renal Doppler ultrasound would have been useful in the earlier stages to make sure that blood flow had stopped to the relevant area; particularly the micro probe would have been useful as it can be left on the kidney to monitor perfusion after renal clamping. To date, we have not used any intra-arterial fluorescence technology which has been used with some success at other centres [14].

For smaller tumours, clamping the artery alone was helpful as venous bleeding as a whole was not a problem and could be dealt with by increasing intra-abdominal pressure. In our experience, renal vein clamping is not necessary unless the tumour is centrally located and large venous branches are opened [15, 16].

Predictably, those patients with very short clamp times under ten minutes did not even have transient changes in serum creatinine, although it would be of value to follow renal function over a more extended period to see whether even a short period of renal ischaemia is damaging in the long term.

Long-term renal function outcomes and long-term oncological outcomes are obviously of the highest importance. While we are not able to report on these here, we will be monitoring them with interest. At the time of reporting none of the patients in the present series had any evidence of recurrence on routine follow-up imaging. A limited calculation based on 19 of our patients showed an average rise in total serum creatinine of 4.9% at an average follow-up of four months. In the literature, intermediate term data has been published with follow-up data at two to six years after RAPN in a group of 134 patients showing effective oncologic control and renal function preservation with an average drop of 8% in eGFR [17].

In terms of positive surgical margins (PSM), comparison between the gold standard of OPN and RAPN has been conducted with a multicentre comparison study of 198 OPN versus 105 RAPN showing no significant differences in PSM rates (5.6 versus 5.7%) [18]. For RAPN, the reported PSM rates vary widely. One series of 220 RAPN reported PSM of 8.2% [19], another series of 153 patients [20] reported PSM of 3.5%, and another series of 134 patients reported PSM of 0.7% [17]. Our series had no frankly positive margins; even if the one patient (1.7%) with lymphovascular invasion at the margin is counted our results are comparable to the literature.

Our overall complication rate of 15.8% is comparable to that achieved in the current standard of literature, with a US analysis of 886 patients undergoing RAPN at 5 US centres coming out with an overall complication rate of 15.6% [21]. However, it should be noted that over 66% of our complications were Clavien I and II and that progression to investigative intervention in two of our other patients did not reveal any complicating pathology. They reported a 4.6% perioperative transfusion rate, compared to our 3.4% transfusion rate, and the most common complication that they reported after RAPN was postoperative haemorrhage which can in most cases be managed conservatively [21].

In summary, the lessons we wish to impart include the following:

- The importance of dedicated CT arteriography to help in detecting a second or third renal artery, and in defining vascular anatomy to allow segmental clamping; as more complex tumour surgery is attempted, it is this segmental clamping that will allow us to avoid global ischaemia to the kidney.
- The essential role of the sliding clip renorrhaphy technique with early unclamping.
- The usefulness of intraoperative ultrasound.
- The importance of having an experienced bedside assistant, preferably a second urologist with laparoscopic skills.

## 5. Conclusion

Use of the Da Vinci robot in assisting laparoscopic partial nephrectomy will allow experienced laparoscopic surgeons to perform the procedure with shorter ischaemic times to the kidney.

This series demonstrates that RAPN is feasible and can be performed safely with similar oncological outcomes to OPN in an Australian setting when performed by experienced laparoscopic surgeons in a dedicated high volume robotic centre.

## Figures and Tables

**Table 1:** Patient and tumour data.

Baseline data	
RAPN procedures (*n*)	77
Number of patients (*n*)	76
RAPN procedures, senior author	58
Mean age, y (range)	63 (36–83)
Sex (*n*)	
Male	41
Female	16

Tumour characteristics (procedures by senior author, *n* = 58)	
Side (*n*)	
Left	33
Right	25
Mean tumour size, mm (range, median)	30.5 (14–80, 28)
Pathology, *n* (percentage)	
Clear cell	36 (62.1%)
Papillary cell	8 (13.8%)
Oncocytoma	7 (12%)
Chromophobe	4 (7%)
Angiomyolipoma	2 (3.4%) (fat-poor)
Malakoplakia	1 (1.7%)
Negative margin status, *n* (%)	57 (98.3%)
Lymphovascular invasion at margin, *n* (%)	1 (1.7%)

**Table 2:** Perioperative data.

Perioperative data (procedures by senior author, *n* = 58)	
Median warm ischemia time, min (range)	8 (0–30)
Median operative time, hours (range)	4 (1.5–6)
Mean (hours)	4.2
Conversion to open, *n* (%)	1 (1.7%)
Intraoperative transfusion, *n* (%)	1 (1.7%)
Postoperative transfusion, *n* (%)	1 (1.7%)
Median length of stay, d (range)	6 (3–23)
Mean (d)	6.8

**Table 3:** Warm ischaemia times achieved by primary operator only (58 patients).

Patient number	Warm ischaemic time (range in minutes)	Warm ischaemic time (median in minutes)
1 to 10	10–30	18
11 to 21	6–20	9
22 to 58^†^	0–20	4.5^†^
Overall	0–30	8

^†^11 of these cases had zero ischaemic time.

**Table 4:** Complications by Clavien grade.

Grade I	(i) Atelectasis in 3 patients^†,‡^ (ii) Pneumothorax, resolved without drain^†^ (iii) Gluteal compartment syndrome^§^

Grade II	(i) Pneumonia (ii) Blood transfusion in 2 patients^‡,||^ (iii) DVT/PE

Grade IIIa	Haematuria requiring angiogram: negative study, haematuria spontaneously resolved

Grade IIIb	(i) Laparoscopy for investigation of acute abdomen, no abnormalities found, likely reperfusion injury (ii) Conversion to open nephrectomy for significant venous bleeding^||^

Grade IV	None

Grade V	None

^†^One patient had both atelectasis and pneumothorax.

^‡^One patient had both blood transfusion and atelectasis.

^§^Conservative management only. Fasciotomy not required.

^||^The patient who underwent conversion to open nephrectomy also received blood transfusion.
